# Highly Selective Near-Infrared Nanosensors for Dopamine Detection: Leveraging the Phase–Engineered ssDNA-SWCNT Nanosensors

**DOI:** 10.1007/s10895-026-04849-7

**Published:** 2026-06-19

**Authors:** Ramazan Bayat, Muhammed Bekmezci, Damla Ikballi, Iskender Isik, Fatih Sen

**Affiliations:** 1 Sen Research Group, Department of Biochemistry, Dumlupinar University, Kutahya, 43000 Türkiye; 2 Department of Materials Science & Engineering, Faculty of Engineering, Dumlupinar University, Kutahya, 43000 Türkiye

**Keywords:** SWCNT, Dopamine, Fluorescence, Sensor, Near infrared region, Molecular identification

## Abstract

Single-walled carbon nanotubes (SWCNTs) that emit fluorescence in the near-infrared region (NIR) enable the identification of certain molecules as a result of surface modification. In this study, a selective sensor system against dopamine, a neurotransmitter, was developed by wrapping a Guanine-Thymine-Adenine (GTA)_20_-repeat single-stranded DNA structure around SWCNT. Sensor analysis fluorescence measurements in the NIR region were performed using a customized microscope system. The fluorescence activity of the developed (GTA)_20_-SWCNT sensor against different biological samples was examined in the NIR region. It was observed that the sensor was selective against dopamine. In the study conducted in the 10–100 µM linear range, it was observed that an increase in fluorescence intensity occurred due to increasing dopamine (DA) concentration. The limit of detection (LOD) of the sensor was calculated as 4.2 × 10^− 5^ M, and the significant limit of quantification (LOQ) was calculated as 13.24 × 10^− 5^ M. With these features, the (GTA)_20_-SWCNT biosensor has the potential to be used as an innovative tool in DA detection.

**Supplementary Information:**

The online version contains supplementary material available at 10.1007/s10895-026-04849-7.

## Introduction

The technology that develops as a result of increasing scientific activities plays a major role in the development of biosensors. Advanced systems are required for early diagnosis of diseases, personalized treatment, and illumination of biological and chemical systems [1]. At the forefront of these systems are, of course, biosensors. Biosensors are tools used in diagnosis in basic research areas such as health, biomedical and environmental [2–4]. Fluorescence sensors, a class of biosensors that are frequently used today, are used to detect analytes quickly, without contact, and in real time [5–9]. In recent years, developments in the field of nanomaterials have led to advances in the field of bıosensors [1, 10, 11]. In particular, an understanding of carbon chemistry has enabled structures such as graphene, carbon quantum dots, and carbon nanotubes to be used as biosensors.

Single-walled carbon nanotubes (SWCNTs) are quite remarkable in innovative nanotechnology fields such as biological imaging, electronics, and with their optoelectronic properties [5, 12–14]. SWCNTs can be called 1D structures formed by folding a single graphene layer in a cylindrical structure. The folding direction determines the crystallinity of SWCNTs and enables the formation of structures with a length of a few nanometers and a diameter of 1–2 nm [15–18]. SWCNTs chiralities in semiconductor structures give fluorescence emission in the near infrared (NIR) range (900–1400 nm) [16, 19–21]. Compared with organic fluorophores, SWCNTs are not affected by photodegradation or blinking, making them suitable sensors for long-term processes [22, 23]. SWCNTs, which has hydrophobic properties, precipitates by agglomeration in an aqueous environment [24]. Surface functionalization is required to prevent aggregation of SWCNTs in aqueous solutions and to preserve and enhance their fluorescence properties [25–27]. The functionalization process allows the identification of various molecules as well as the formation of colloidal SWCNTs. Surface functionalization using polymers creates pockets that selectively interact with analytes, and this process is called corona phase molecular identification [15]. As a result of the excitation of SWCNTs from the light source, excitons allow movement along 100 nm. In various studies, many different materials such as surfactants, ssDNA, RNA, proteins, peptides, amino acids and amphiphilic polymers have been used to colloidalize and functionalize SWCNTs [18]. Conjugation of the semiconducting SWCNTs surface with polymers has enabled the formation of a new class of bıosensors [28]. Functionalization of the surface of SWCNTs with materials such as polymers and interactions with their immediate environment can affect the NIR fluorescence emission. Wavelength shifts or changes in fluorescence intensity resulting from the NIR emission enable SWCNTs to be used as an optical sensor in chemical sensing or imaging [12, 25, 29]. Especially, SWCNTs formed with ssDNA corona phase have started to be used frequently in the detection of analytes. In the SWCNT-ssDNA structure, the ssDNA sequence is responsible for the analyte-specific molecular recognition, which has led to the extensive study of ssDNAs [13, 30]. SWCNTs provides fluorescence emission in the NIR region, low background noise and high penetration in biological tissues, providing a great advantage in imaging tissues. The functionalisation of SWCNTs surfaces with polymers or different molecules has enabled the detection of hydrogen peroxide [31], cholinesterase enzyme activity [32], nitric oxide [23, 33], glucose [34] at the single molecule level. It is hypothesised that corona phase molecular recognition will facilitate the identification of a multitude of molecules and enable the discovery of novel materials. Monitoring and understanding human health requires the accurate analysis of various physiological and psychological parameters. The detection of biomolecules in body fluids is considered a significant technological advancement in the field of health diagnosis [35].

Neurotransmitters are integral to chemical signalling in the human body, particularly within the neuronal network of the brain [15, 28, 36]. Dopamine is a key catecholamine neurotransmitter in the central nervous system and has versatile functions [37]. Dopamine is also associated with psychiatric disorders such as Parkinson’s disease and Alzheimer’s disease and is closely related to the central nervous system [38, 39]. Although dopamine (DA) is present in the human body, its concentration is generally quite low under physiological conditions and is typically below 100 nM in most tissues [37]. Currently, electrochemical detection [40], chromatography [41], spectroscopy [42], and enzyme-linked immunosorbent assay are frequently used for DA detection [37, 43]. While each of these techniques used today has its own advantages, they also have significant limitations; for example, the preparation of electrochemical sensors often involves complex processes and there is a problem of low spatial resolution [44]. Optical sensors, on the other hand, present various issues in terms of tissue penetration, selectivity, and stability [37, 45]. Selective and stable methods need to be developed to address the problems encountered in DA detection, in line with advancing technology. The neurotransmitter dopamine plays a role in regulating both motivation and motor function. A significant yet challenging objective is to gain a deeper comprehension of cell-to-cell communication, which entails determining the temporal and spatial resolution of dopamine and other neurotransmitters [46]. SWCNT-based sensors provide very high temporal and spatial resolution for imaging neurotransmitters, in contrast to other techniques such as electrochemical or genetically encoded sensors [15].

In this study, a new generation fluorescence dopamine biosensor was developed by monitoring the behaviour of dopamine in the NIR region using SWCNT-wrapped single-stranded d(GTA)_20_ oligonucleotide (GTA)_20_-SWCNT). In this study, a customized microscope system incorporating an NIR camera, laser, and filter system was used to develop a selective sensor and sensor system for detecting dopamine, a neurotransmitter, using the developed biosensor. The results of the study demonstrated that SWCNT-based optical systems can be used without the need for expensive systems.

## Materials and Methods

### Preparation of (GTA)_20_-SWCNT Sensor

(GTA)_20_-SWCNT biosensor prepared using ssDNA (GTA)_20_ was performed using the previously published method [23, 33, 47]. Firstly, 2 mg ssDNA (GTA)_20_ was dissolved in 0.1 M NaCl, and 1 mg HIPCO SWCNT (Unidym) was added. The prepared sample was sonicated (Bandeline Sonoplus HD 3200) at 40% amplitude for 10 min in an ice bath using a 1/8” titanium probe. The solution was then centrifuged at 16,000 g for 90 min at 25 °C (Sigma 3–30 KS), and approximately 90% of the supernatant was collected for use in sensor experiments.

### Single Molecule Detection

**Scheme 1:**
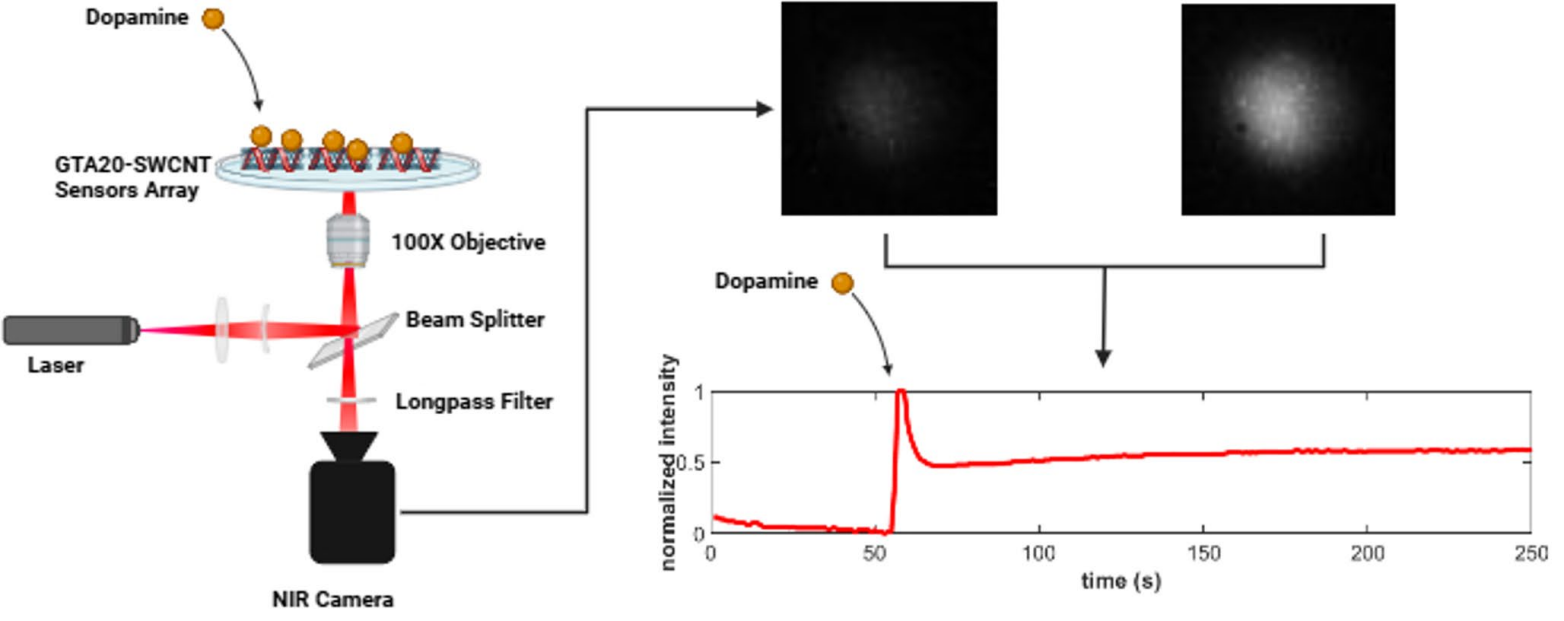
Schematic representation of the acquisition of measurements in the NIR region using (GTA)_20_-SWCNT (Created in https://BioRender.com)

In order to perform single-molecule detection, a (GTA)_20_-SWCNT film was formed on the surface of a glass-bottom petri dish (Matsunami, D35-27-1.5-U). Firstly, 200 µL of 10% (3-Aminopropyl)triethoxysilane (APTES) was dropped onto the surface of the glass-bottom petri dish and quickly washed three times with 1X Phosphate-Buffered Saline (PBS) (Bioshop). Then, a drop of (GTA)_20_-SWCNT solution was added and held for 3 min. The specimen was washed three times with 1X PBS rapidly and efficiently and subsequently stored at room temperature for 10 min [33, 48]. Subsequently, the requisite measurements were conducted in 1X PBS (pH:7.4) medium (GTA)_20_-SWCNT sensor structure measurements were performed in glass-bottomed Petri dishes at room temperature. First, 1X PBS was added, and then the measurement was started. The analyte was added at the 60th second of the measurement, and the measurement continued until the 300th second. This process was performed separately for each analyte. The measurements in the near-infrared (NIR) region were conducted in accordance with the methodology outlined in Scheme 1. In this context, measurements were performed using a modified microscope system (Zeiss Axio Observer 3) with a 100X TIRF objective (Zeiss), a 40 mW 660 nm laser (Omicron Luxx 660 − 130), a dichroic beamsplitter (Chroma T760lpxr-UF1) and an NIR camera (Artcam-008TNIR). The movies recorded at 0.78 f/s were, and the 50 × 50 pixel areas in the obtained movie files were subjected to analysis using MATLAB codes. The fluorescence emission changes of SWCNT films wrapped with (GTA)_20_ were calculated using Eq. 1.

1 $$[(I-I_0)/I_0]^*100$$

In the equation, I represents the final fluorescence intensity, and I_0_ represents the initial fluorescence intensity [23, 33]. The limit of detection (LOD) and limit of quantification (LOQ) values for the tested DA were calculated using equations (Eq. 2) and (Eq. 3).

2 $$LOD=3.3s/m$$

3 $$LOQ=10s/m$$

Here, ‘s’ represents the standard deviation of the y-intercept of the regression line and ‘m’ represents the slope of the calibration curve.

## Result and Discussion

**Fig. 1:**
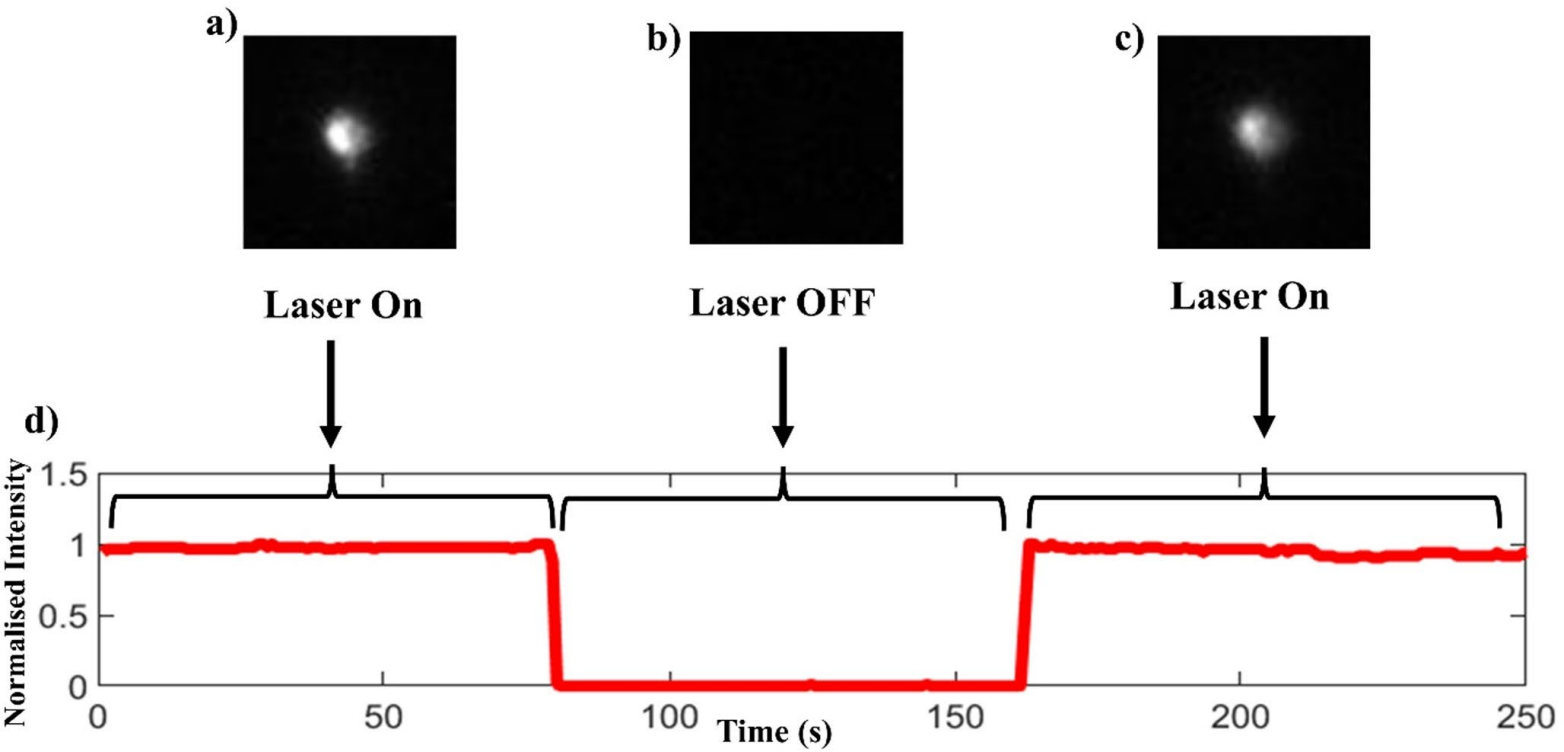
Fluorescence activity graph (**d**) in glass-bottom petri dishes with (GTA)_20_-SWCNT films in laser on (**a** and **c**) and laser off (**b**) conditions

Previous studies on dopamine-targeted SWCNTs have shown that repeating sequences of the bases guanine (G) and thymine (T) (i.e., poly-(GT) sequences) provide high sensitivity and selectivity for this neurotransmitter [5, 15]. Therefore, the (GTA)_20_ DNA sequence was used in this study. (GTA)_20_ was wrapped around SWCNT and then characterized using UV-VIS-NIR adsorption spectroscopy (Figure S1). Looking at the UV-VIS spectrum of (GTA)_20_, an absorption peak belonging to the DNA molecule is observed at 260 nm (Figure S1a) [49]. The absence of other peaks indicates that the DNA is pure. As shown in Figure S1 b, SWCNT exhibits the highest adsorption peak at 290 nm, while a sharp adsorption peak of (GTA)_20_ is observed after the formation of the corona-phase structure. Adsorption studies of SWCNT and (GTA)_20_-SWCNT were performed in the NIR region (Figure S1 c). Wrapping around the SWCNT, (GTA)_20_ has caused slippage at the peak points. This indicates that a DNA-SWCNT structure has been formed [33]. Fluorescence measurements in the near-infrared (NIR) region were conducted using (GTA)_20_-SWCNTs, employing the system depicted in Scheme 1. The time-dependent change data were collected in glass-bottom petri dishes containing 1X phosphate-buffered saline (PBS). Excitation of the SWCNTs was achieved through the utilisation of a 35 mW 660 nm laser, resulting in the generation of fluorescence within the NIR region. As shown in Fig. 1, SWCNTs exhibit fluorescent emission when stimulated by a laser (Fig. 1a), whereas no fluorescent emission occurs in the absence of laser stimulation (Fig. 1b). Subsequently, it was observed that fluorescent emission reappeared upon reactivation of the laser (Fig. 1c). Upon activation and deactivation of the laser, it was observed that fluorescence could be produced as a consequence of excitation. Figure 1d shows any selected point within the selected general area.

**Fig. 2:**
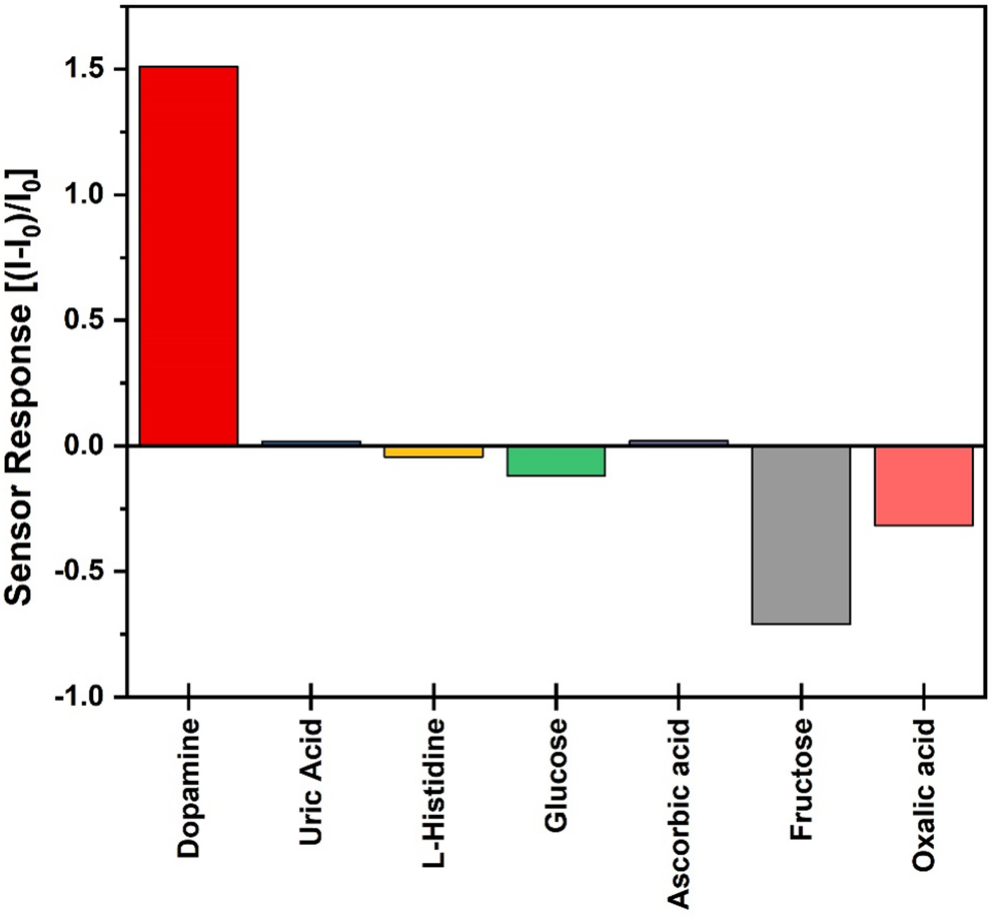
Fluorescence change of (GTA)_20_-SWCNT in the presence of different biological molecules

The (GTA)_20_-SWCNT biosensor solution was then coated on the surface of a glass-bottom petri dish using a customized microscope to form a film for NIR fluorescence analysis in the presence of biological samples separately. As illustrated in Fig. 2, the selectivity map demonstrates the behaviour of diverse molecules in the NIR region when exposed to 50 µM, as determined through corona phase molecular recognition.

An examination of the selectivity map reveals that the fluorescence change in the presence of uric acid, L-histidine, glucose, L-ascorbic acid, and oxalic acid molecules is relatively modest. In contrast, the presence of fructose results in partial quenching of fluorescence intensity, while the presence of dopamine leads to an increase in fluorescence intensity.

**Fig. 3:**
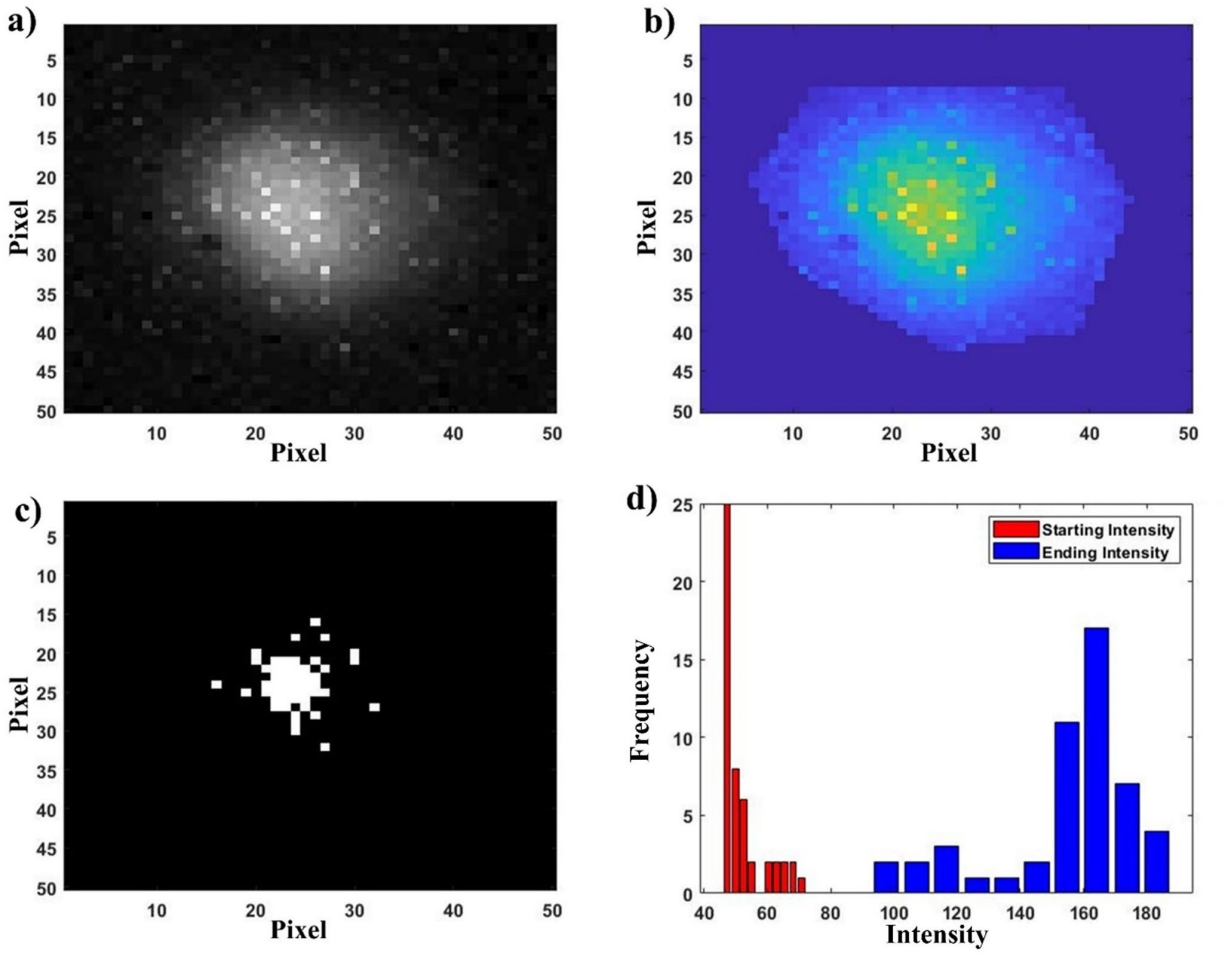
Fluorescence analysis of (GTA)_20_-SWCNT in the presence of 100 µM dopamine. (**a**) First frame of the acquired image. (**b**) Shows the cropping points of the image. (**c**) 50 brightest selected points. (**d**) Frequency distributions of starting and ending fluorescence

As in previous studies, an increase in fluorescence intensity has been observed in the presence of DA in SWCNTs coated with ssDNA [28]. The molecular dynamics simulations revealed that the interaction between the polar groups of dopamine and the phosphate backbone of DNA brings the backbone closer to the SWCNT, which in turn creates a change in the electrostatic potential on the SWCNT surface [15]. The increase in luminescence intensity observed in the presence of DA (commonly referred to as the “turn-on response”) is the result of complex photophysical and molecular recognition mechanisms [28]. The potential for (GTA)_20_-SWCNT selectivity, redox selectivity, non-radiative energy loss, and steric hindrance factors all play a role in this process [6, 23, 27]. In particular, the DNA helical structure formed around SWCNTs has the effect of partially blocking the fluorescence radiation of SWCNTs. Dopamine can facilitate the filling of gaps at the LUMO level of DNA by providing electrons at the HOMO level [23]. The collection of data of (GTA)_20_-SWCNT using the customised microscope and analysis with MATLAB is shown in Fig. 3. Figure 3a shows the first video frame of the measurement. In the present study, the measurements are performed regarding the first video frame. Therefore, the sensor structure must remain constant throughout the measurement [50]. The initial video frame was selected and cropped to identify the brightest points within the frame (Fig. 3b). The 50 brightest spots were selected from the cropped region and employed in the subsequent measurements (Fig. 3c). Figure 3d illustrates the initial and final fluorescence intensity frequency distributions of the 50 brightest spots. As can be observed, an increase in fluorescence intensity was evident at the conclusion of the experiment, which was attributed to the addition of dopamine.

**Fig. 4:**
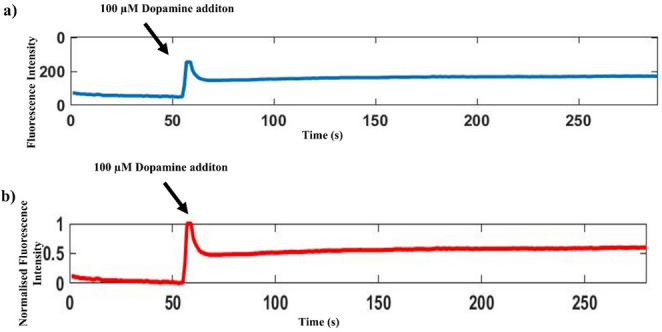
Time-dependent fluorescence intensity (**a**) and normalized fluorescence intensity (**b**) change after dopamine addition to the (GTA)_20_-SWCNT sensor

The dopamine molecule acts to prevent the loss of energy in SWCNTs by modifying the interaction of SWCNTs with (GTA)_20_. This results in an increase in the fluorescence of the SWCNT in the presence of dopamine. This is achieved by the electrons from dopamine replacing those of the SWCNTs [23]. Figure 4 shows the fluorescence intensity (Fig. 4a) and normalized fluorescence intensity (Fig. 4b) time-dependent fluorescence changes of a randomly selected spot. As can be seen, the addition of dopamine resulted in an increase in fluorescence intensity. The changes in fluorescence intensity were analysed separately for the 50 brightest spots. As can be seen in the non-normalised and normalised time-dependent fluorescence changes graphs shown in Figure S2 and Figure S3, similar fluorescence change graphs were exhibited at each point. The addition of dopamine resulted in an increase in fluorescence intensity at the same time at each point.

**Fig. 5:**
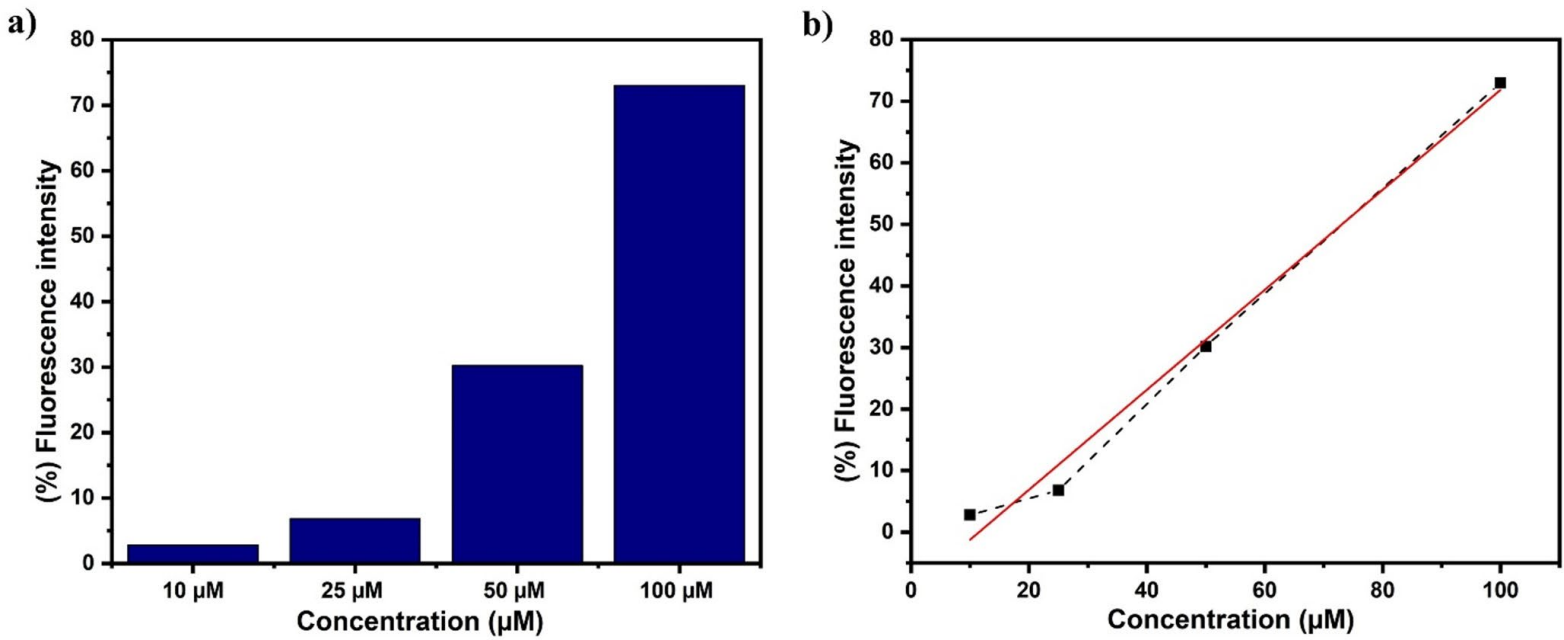
Fluorescence change (**a**) and calibration graph (**b**) of the (GTA)_20_-SWCNT sensor after dopamine addition at different ratios

The fluorescence activity of the (GTA)_20_-SWCNT sensor in the presence of different concentrations of dopamine was investigated (Fig. 5). Studies show that the fluorescence change is dependent on the concentration of dopamine (Fig. 5a). The calibration graph in Fig. 5b shows that the fluorescence intensity increases linearly with increasing concentration [15, 36].

R^2^=0.9741 was found in the calibration graph. The high R^2^ value indicates that the results are significant. The increase in fluorescence intensity due to the increase in concentration indicates that (GTA)_20_-SWCNT can be used as a sensor against dopamine. As a result of the studies carried out in this context, LOD and LOQ values were calculated as 4.2 × 10^− 5^ M and 13.24 × 10^− 5^ M, respectively.

The LOD value in the sensor structure developed using (GTA)_20_-SWCNT against DA is seen to be above the value accepted in the literature. The main reason for this is the low resolution of the NIR camera. The low resolution of the camera has limited the ability to obtain results at low concentrations. To overcome this situation, more advanced camera systems must be used [15, 28, 51, 52]. The sensor system developed against dopamine using (GTA)_20_-SWCNT offers a new perspective for the detection of dopamine.

## Conclusion

In this study, a dopamine-sensitive biosensor capable of operating in NIR was developed by utilizing the semiconducting properties of SWCNTs. The performance of the developed (GTA)₂₀-SWCNT nanosensor structure was evaluated using different analytes, and its selectivity profile was determined. The analysis of the 50 brightest spots selected in the images taken in the NIR region revealed that each spot exhibited a comparable fluorescence change profile in the presence of dopamine. From the graph generated from the dopamine measurements conducted at varying concentrations, LOD and LOQ values were calculated as 4.2 × 10^− 5^ M and 13.24 × 10⁻⁵ M, respectively. The findings suggest that (GTA)_20_-SWCNT may be an alternative method for dopamine detection. This study offers the potential for SWCNTs, which emit fluorescence in the near-infrared region, to be detected in a novel manner for the identification of diverse molecules as a consequence of surface modification.

## Supplementary Information

Below is the link to the electronic supplementary material.

Supplementary Material 1 (DOCX 2.23 MB)

## Data Availability

No datasets were generated or analysed during the current study.
