# Supplementary material for: Highly Selective Near-Infrared Nanosensors for Dopamine Detection: Leveraging the Phase–Engineered ssDNA-SWCNT Nanosensors
Source: J Fluoresc. 2026 Jun 19;36(6):4181–8. doi: 10.1007/s10895-026-04849-7 (PMC13331960; doi:10.1007/s10895-026-04849-7)
Supplement: Supplementary file 1 — Supplementary Material 1 (DOCX 2.23 MB) [file 10895_2026_4849_MOESM1_ESM.docx]

**Highly Selective Near-Infrared Nanosensors for Dopamine Detection: Leveraging the Phase–Engineered ssDNA-SWCNT Nanosensors**

**Ramazan Bayat ^a,b^, Muhammed Bekmezci ^a,b^, Iskender Isik ^b^, Fatih Sen^a*^**

*^a^ Sen Research Group, Department of Biochemistry, Dumlupinar University, 43000, Kutahya, Türkiye*

*^b^ Department of Materials Science & Engineering, Faculty of Engineering, Dumlupinar University, Kutahya, 43000, Türkiye*

**Supporting Information**


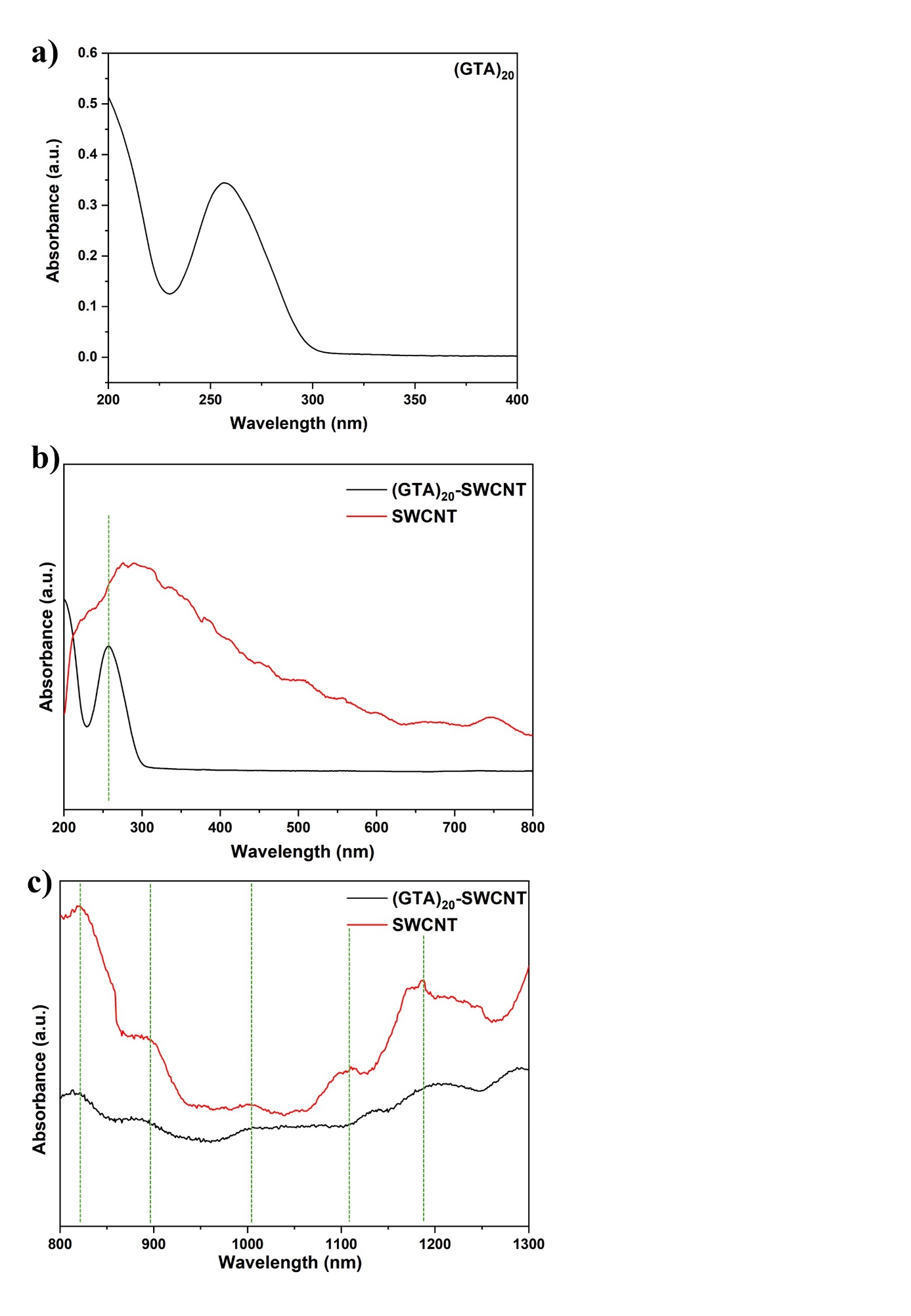


**Figure S1.** UV-VIS absorption spectrum of (GTA)_20_ (a), (GTA)_20_-SWCNT and NIR absorpsiyon spectrum of (GTA)_20_-SWCNT (c) suspension.


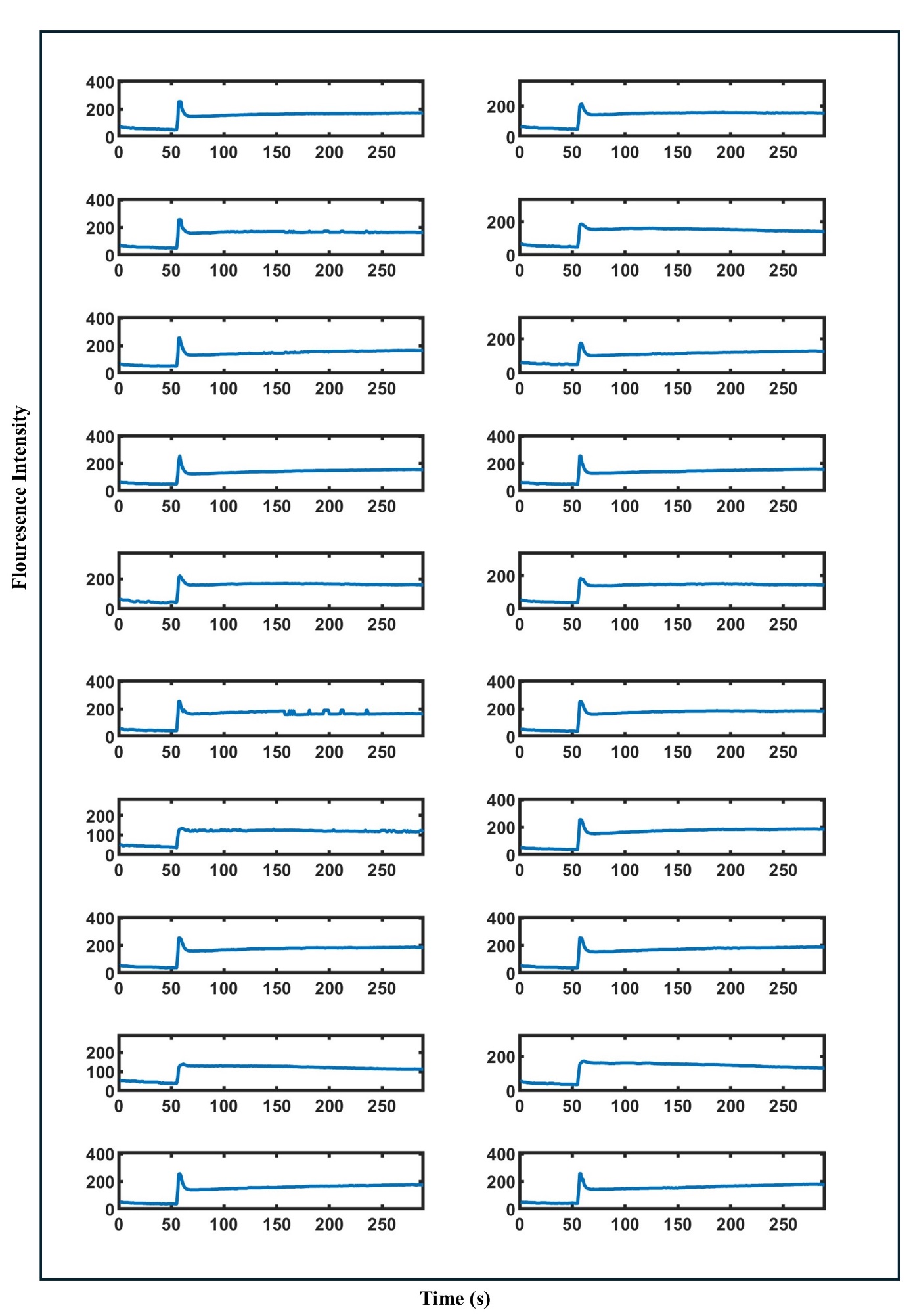


**
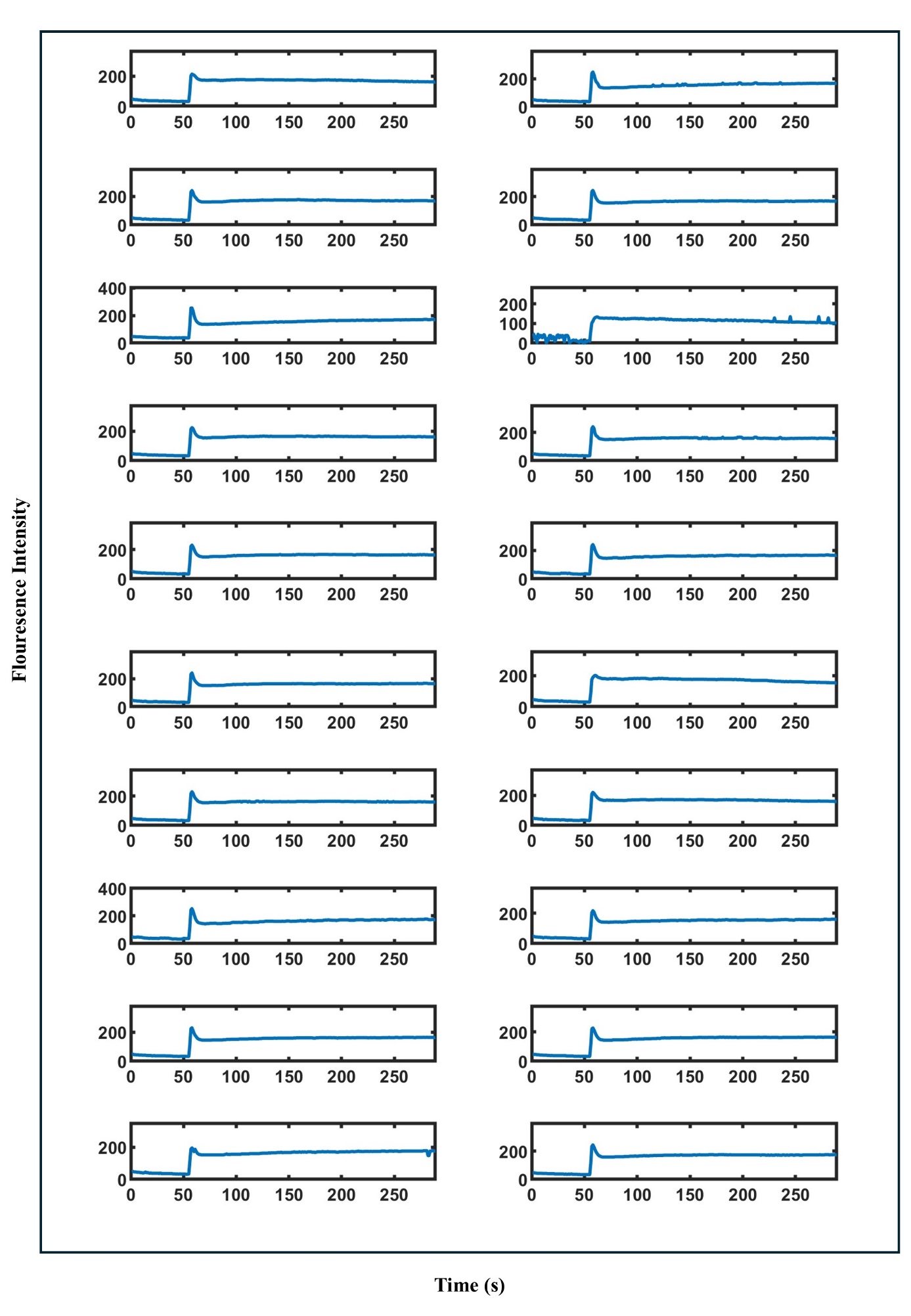
**

**
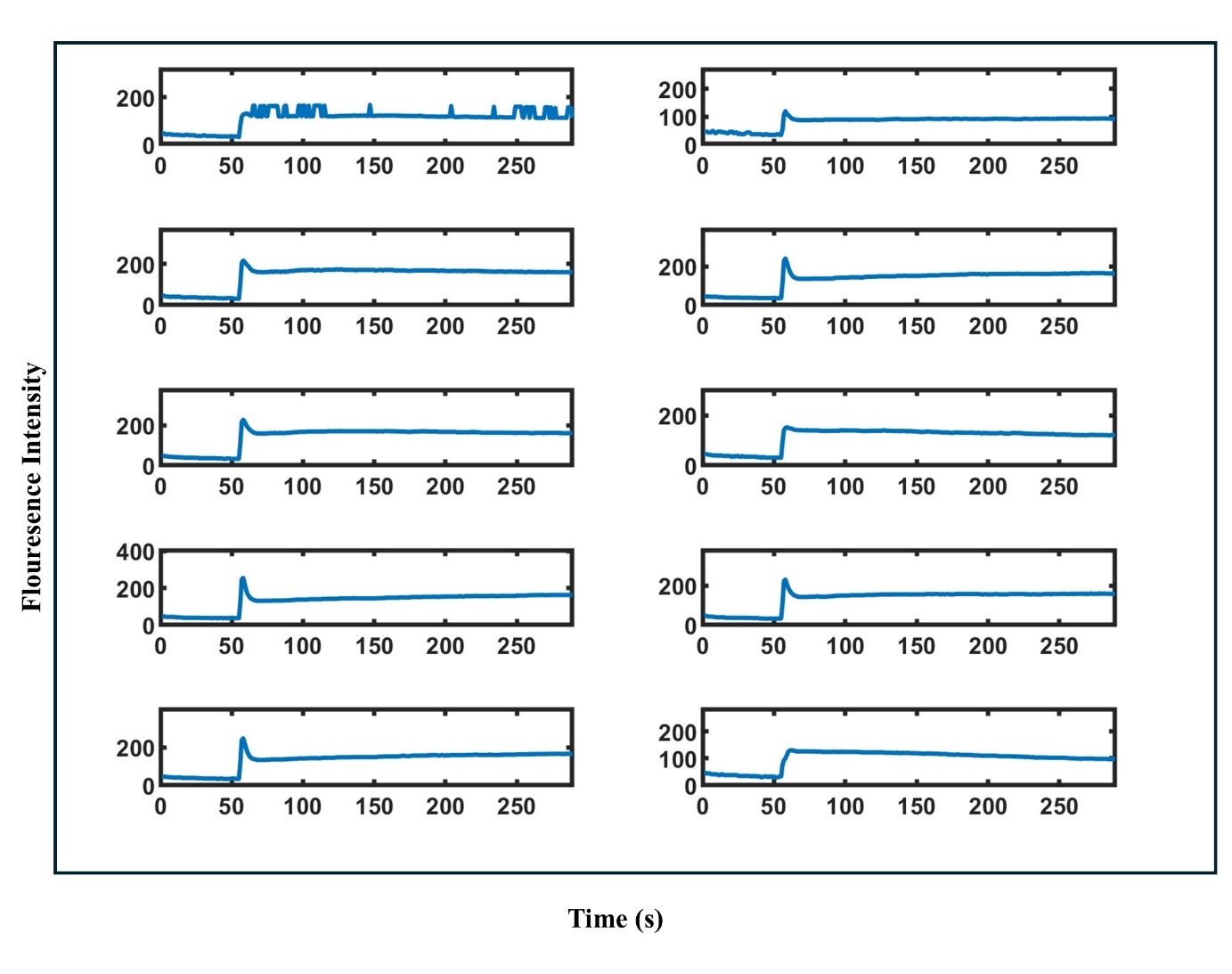
**

**Figure S2.** Time-dependent fluorescence change of the 50 brightest spots on the (GTA)_20_-SWCNT sensor after dopamine addition.


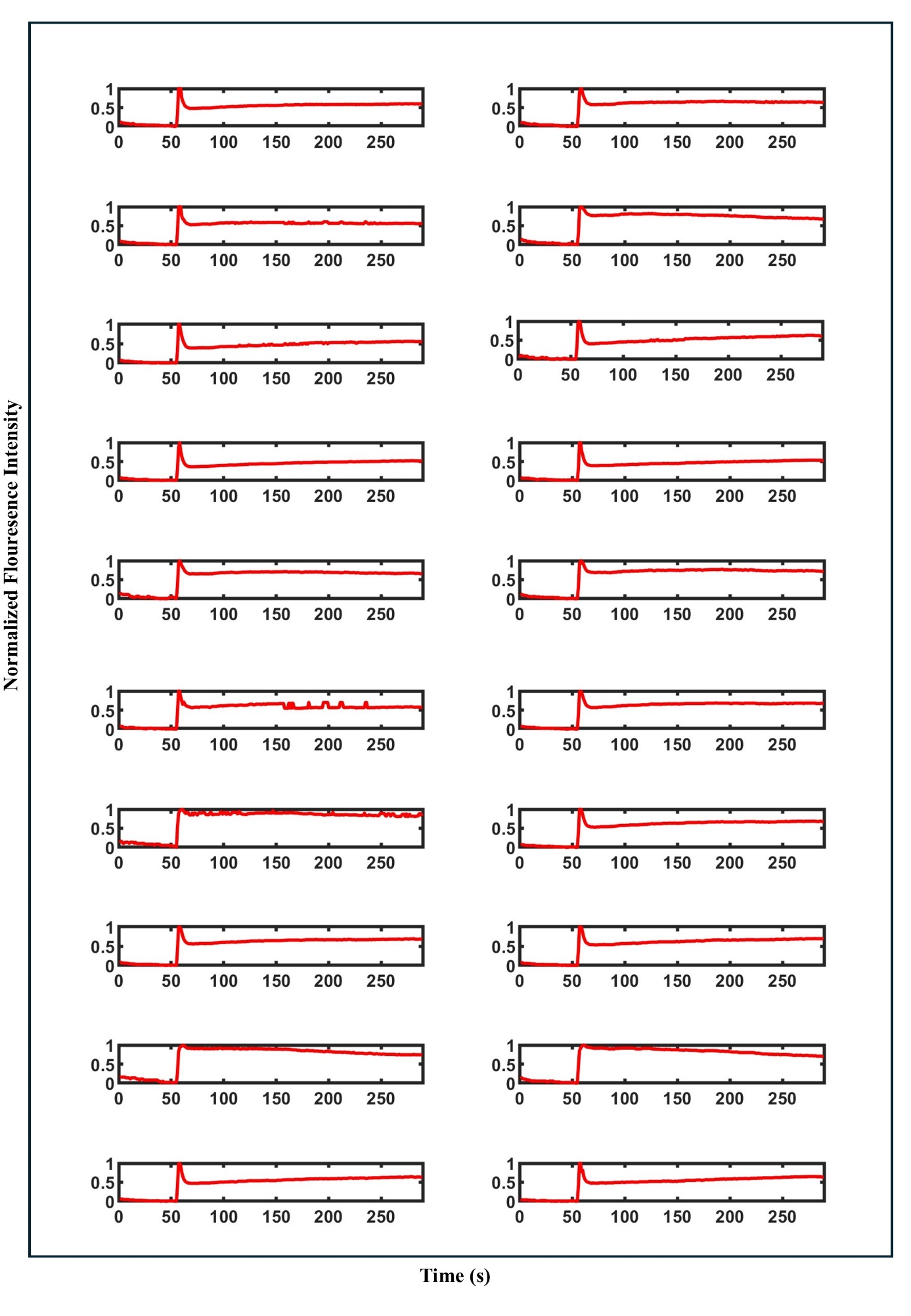


**
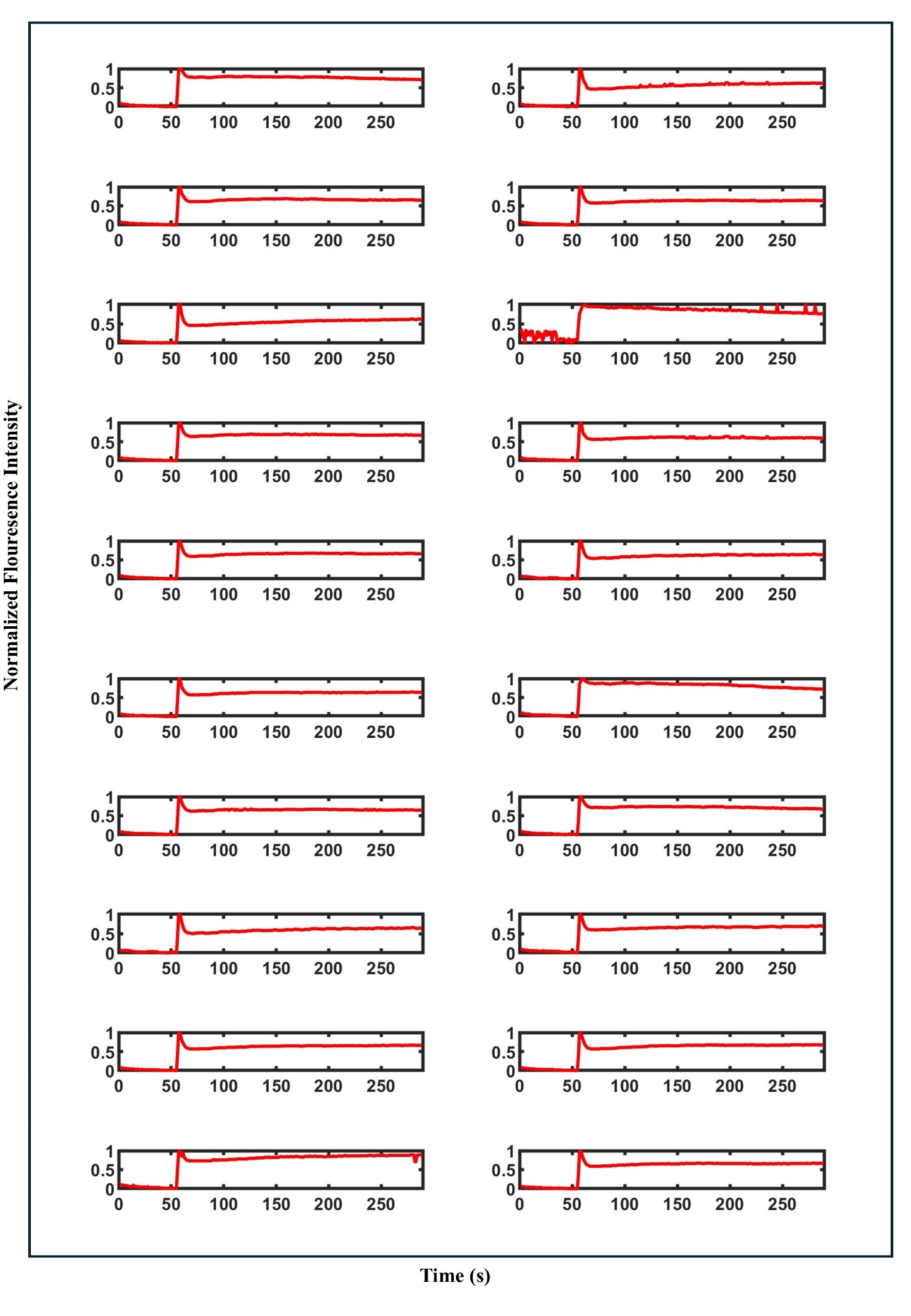
**

**
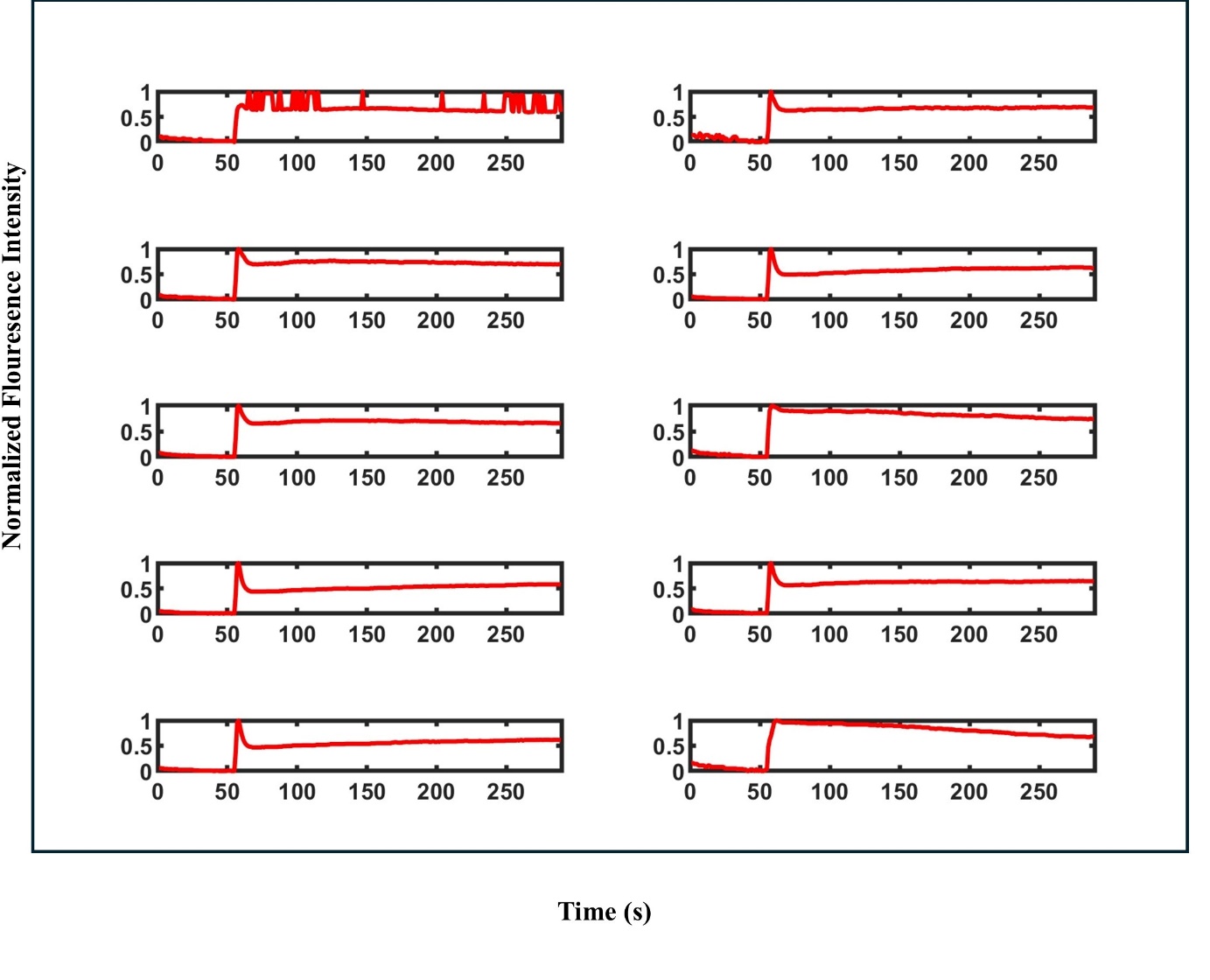
**

**Figure S3.** Time-dependent normalized fluorescence change of the 50 brightest spots on the (GTA)_20_-SWCNT sensor after dopamine addition.
